# Associations between maternal BMI, breastfeeding practices and infant anthropometric status in Colombia; secondary analysis of ENSIN 2010

**DOI:** 10.1186/s12889-020-8310-z

**Published:** 2020-02-14

**Authors:** Fanny Aldana-Parra, Gilma Olaya Vega, Mary Fewtrell

**Affiliations:** 1 Departamento de Nutrición y Bioquímica, Pontificia Universidad Javeriana, Bogotá, Colombia; 2 GOS Institute of Child Health, University College London, London, UK

**Keywords:** Maternal nutritional status, Lactating women, Infant nutritional status, Maternal-infant health, Colombian population, Stunting, Wasting, Maternal overweight, Maternal undernutrition, Children overweight, Breastfeeding, Exclusive breastfeeding

## Abstract

**Background:**

Maternal malnutrition and infant feeding mode impact short and long term infant and child morbidity and mortality. The period of lactation may provide an opportunity to modulate the risk of disease later in life. Our aim was to estimate the effect of maternal body mass index (BMI) and infant feeding mode, particularly breastfeeding practices, on the anthropometric status of children under 2 years in Colombia.

**Methods:**

A secondary analysis was performed using the data from ENSIN 2010. Term infants under 2y, singleton, with a mother older than 18y, were included in the analysis. Outcomes were wasting (WLZ < -2SD), overweight (WLZ > +2SD) and stunting (LAZ < -2SD). Predictors were infant feeding (exclusive and predominant BF constructed from 24-h recall, age at introduction of liquids, semisolids and solids) and maternal BMI. Socioeconomic variables, maternal education and age, conditions during pregnancy and birth weight were analyzed as covariates.

**Results:**

Mothers of overweight infants had higher BMI (Mean dif = 1.47 kg/m^2^; 95% CI = 2.1, 0.8) than those with normal weight infants. Stunting and wasting were not predicted by maternal anthropometry or infant feeding mode. Fewer maternal years of education were associated with wasting (OR = 0.90; 95% CI = 0.86, 0.97; *p* = 0.003) and stunting (OR = 0.92; 95% CI = 0.89, 0.94; *p* < 0.0001), while more maternal years of education were associated with overweight (OR = 1.06; 95% CI = 1.02, 1.01; *p* = 0.001); higher birth weight was associated with overweight (OR = 1.001; 95% CI = 1.00, 1.001; *p* < 0.0001) and lower birth was associated with stunting (OR = 0.99; 95% CI = 0.89; *p* < 0.0001) in the final regression model.

**Conclusions:**

Maternal BMI is a modifiable target for public health policy to promote healthy infant growth. Infant nutritional status is affected by direct and indirect factors that need to be addressed in further studies.

## Background

Maternal malnutrition negatively impacts infant and child morbidity and mortality. The Developmental Origins of Health and Disease Hypothesis considers the periconceptional period as a window of opportunity to influence long term body composition and endocrine characteristics of the offspring [1, 2]. Early human observational studies suggested that maternal famine, especially during periods of war, increases the risk of cardiometabolic diseases in the offspring as adults [3]. Maternal obesity could also have a detrimental effect on the development of obesity in offspring, as shown in observational studies [4].

The period of lactation may provide an opportunity to modulate the impact of genetic and prenatal factors experienced by the fetus [5]. Animal models show that pups fed with a cafeteria diet during gestation and lactation had increased adiposity at weaning, which could predispose to metabolic disorders later in life [6]. In humans, formula feeding during the first months of life can result in accelerated weight gain. In a large population study (*n* = 5560), children formula-fed since birth were more likely to be obese at school age (OR = 1.57; 95% CI 1.2–2.2) [7]. Breastfeeding (BF) is associated with protection against respiratory and gastrointestinal infections [8], a positive effect on cognitive and neurological development [9] and reduction of breast, endometrial and ovarian cancer for the mother [10–12]. Other possible but not well-established benefits for the infant include a lower risk of obesity, diabetes [13] and hypertension [14]. However, EBF prevalence during the first 6 months in middle-income countries is around 37% [15], and in Colombia, this decreased from 46.9% in 2005 to 36.1% in 2015 [16].

While global mortality in children under 5 years fell from 93 per 1000 live births in 1990 to 41 per 1000 live births in 2016 [17], and the prevalence of stunting decreased from 39.2% in 1990 to 21.9% in 2018 [18], the prevalence of obesity increased in childbearing women and children under 5 years. In 2015, the worldwide prevalence of obesity in women over 18 years was 15.2%, and in children under 5 years, 5.9% were overweight, 22% stunted and 7.5% wasted during 2017 [19]. During 2015, Colombia, a Latin-American country ranked by World Bank in the upper-middle-income group [20], reported a prevalence of obesity in childbearing women and children under 5 years of 22.4 and 6.3%, respectively and a prevalence of stunting of 10.8% and wasting of 2.3% during the same year (Encuesta Nacional de la Situación Nutricional (ENSIN)) [16].

Considering maternal nutritional status and breastfeeding practices as potential factors that could influence children nutritional status [21, 22], and given the lack of information in Colombia about the associations between these factors and infant stunting, wasting and overweight, the purpose of this secondary analysis using data from ENSIN 2010 was to estimate the effect of maternal body mass index (BMI) and infant feeding mode, particularly breastfeeding (BF) practices, on the anthropometric status of children under 2 years in Colombia.

## Methods

### Study design

This study was a secondary analysis of a national survey conducted in Colombia during 2010: Encuesta Nacional de la Situación Nutricional en Colombia (ENSIN) [23]. The ENSIN is a survey of households with national coverage and urban and rural representation, which includes sociodemographic characteristics of the households, mother and infant anthropometric data, and information about breastfeeding and early feeding. The dataset was provided by the program Demographic Health Survey (DHS) belonging to the United States Agency for International Development (USAID), which aims to collect, analyze and disseminate accurate and representative data on the characteristics of the population, health and nutrition in countries around the world.

### Population and sample

The population of Colombia during 2010 was about 45,510,000 based on estimates from the national population census (*n* = 23,277,201 female; 50.69%) [24]. The ENSIN 2010 is a descriptive and cross-sectional survey that describes the Colombian sociodemographic and nutritional situation, in a population from birth to 64 years old in 258 municipalities, both rural and urban. The ENSIN sample was randomly selected from the national census of 2005, whose calculation was based on the estimation of global malnutrition according to previous national surveys (ENSIN and ENDS 2005) [25]; the methodological design of ENSIN 2010 is described elsewhere [23]. It included 17,756 children under 5 years. For the present analysis, inclusion and exclusion criteria were:

- Inclusion criteria: Infant under 2 years (*n* = 7002) with gestational age at birth ≥37 weeks.
- Exclusion criteria: Infant from multiple pregnancy (*n* = 100; 1.43%), pregnancy during the survey (*n* = 250; 3.57%), gestational age at birth less than 36 weeks (*n* = 703; 10.03%) and infant with implausible weight and/or length (±5 SD of weight for length) (*n* = 248; 4.33%).

Mothers above 18 years who were not pregnant at the time the survey and who were the respondent of the survey were included. If more than one child under 5y was living in a single house, the youngest was included in the survey.

### Data collection

Baseline characteristics, maternal and infant anthropometry and BF and other infant feeding mode data were collected using validated questionnaires in the house of the interviewee. Trained dietitians administered the questionnaires and made infant and maternal anthropometric measurements; all data collection procedures and performance of the interviewers were tested in a pilot study and standardized through procedure manuals. Dietitians participated in a two-month training course for data collection including administration of the questionnaires. The dietitian recorded data in the participant’s house using a portable device with CSPro software, which can detect inconsistencies. The mother provided information about breastfeeding for the youngest child aged under 5y living in the house.

- Measurement of anthropometry
  Infant and maternal weight were measured with a SECA 872 digital scale (capacity of 200 kg and accuracy of 50 g), with minimum clothing or other items that could alter the weight as recommended by WHO [26]. Infant and mother were weighed together, and infant weight was calculated by subtracting the weight of both minus the maternal weight. Maternal height (standing) and infant supine length were taken with portable wooden column scale, suitable for infants and adults, without shoes or ornaments on the head that could interfere with the measurement [26].
- Measurement of predictors of infant anthropometry
  Information about breastfeeding practices during the last 24 h was recorded using a questionnaire. For the present analysis, we selected only information about infants under 2 years.

### Study variables

- Outcome: Infant anthropometry
  Weight for length Z-score (WLZ) and length for age Z-score (LAZ) reflect infant anthropometric status and were calculated using the WHO growth standards [27]. For the present study, infant stunting was defined as LAZ < -2 Standard Deviations (SD), wasting as WLZ < -2 SD and overweight as WLZ > + 2 SD as recommended by WHO [28].
- Predictors of infant anthropometry
  Maternal anthropometric status. Maternal BMI was calculated as the ratio between maternal weight in kg divided by maternal height squared, and analysed as a continuous variable.
  Breastfeeding practices and infant feeding mode variables were evaluated based on WHO indicators: age at initiation of BF, age at introduction of other liquids, semi-solid and solid foods, duration of BF, EBF and predominant BF [29]. EBF was defined as the infant currently being BF and not having consumed other liquids, semisolid or solid food in the last 24 h; EBF information was only available for infants aged under 6 months at the time of the survey. Predominant BF was defined as infants currently BF with consumption of other liquids less than three times in the last 24 h, excluding infants with EBF. Duration of BF was recorded for infants who had already stopped BF. Inconsistencies in infant feeding data, for example, a duration of BF longer than the age of the infant at the time of the survey, were registered as missing values. We considered infant formula feeding separately as a baseline characteristic and as a possible covariate in analyses predicting infant anthropometry.
- Other variables:
  Sociodemographic variables such as place of residence (urban/rural) and wealth index (poorer/poorest, middle and richer/richest) [30] were recorded. Other maternal variables were maternal age, ethnicity (mestizo/minorities), education attainment in years, living with the partner, currently working, alcohol and cigarettes consumption during pregnancy and delivery by caesarean section. Infant variables also included birth weight, gender and age in months. Paternal anthropometric status and parental adiposity, which have been reported to be associated with infant nutritional status in other studies [31, 32], were not analysed due to a lack of information in the survey. ENSIN 2010 did not specify if the mother’s current partner was the father of the infant.

### Statistical analysis

Descriptive analysis of baseline characteristics is presented as prevalences for categorical variables and mean and standard deviation for continuous variables; WLZ was categorized as wasting, normal and overweight and LAZ as stunting or normal. Comparisons between groups were made with t-test, ANOVA, Kruskal Wallis, U-Mann Witney or χ^2^ test as appropriate and the OR and mean difference with the corresponding 95% CI were given. For variables with more than three categories pairwise post hoc comparisons were made using the Bonferroni test. Binary and multinomial logistic regression models were used to examine predictors of wasting, overweight and stunting. The univariate analysis identified significant associations associations of maternal BMI and/or infant feeding mode variables with wasting, overweight and stunting; and then, these were included in the multivariable models (unadjusted, and then subsequently also adjusted for the other covariates). All comparisons and regressions had normal WLZ and LAZ as the reference group. Statistical analysis was performed with IBM SPSS version 24.

### Ethical aspects

Resolution 8430 addresses ethical research aspects in Colombia. All families participating in the ENSIN 2010 gave informed consent after receiving information about survey [23]. For this analysis, the permits were requested from the Instituto Colombiano de Bienestar Familiar (ICBF) and the DHS program for the use of the databases necessary for the analysis.

## Results

**Fig. 1:**
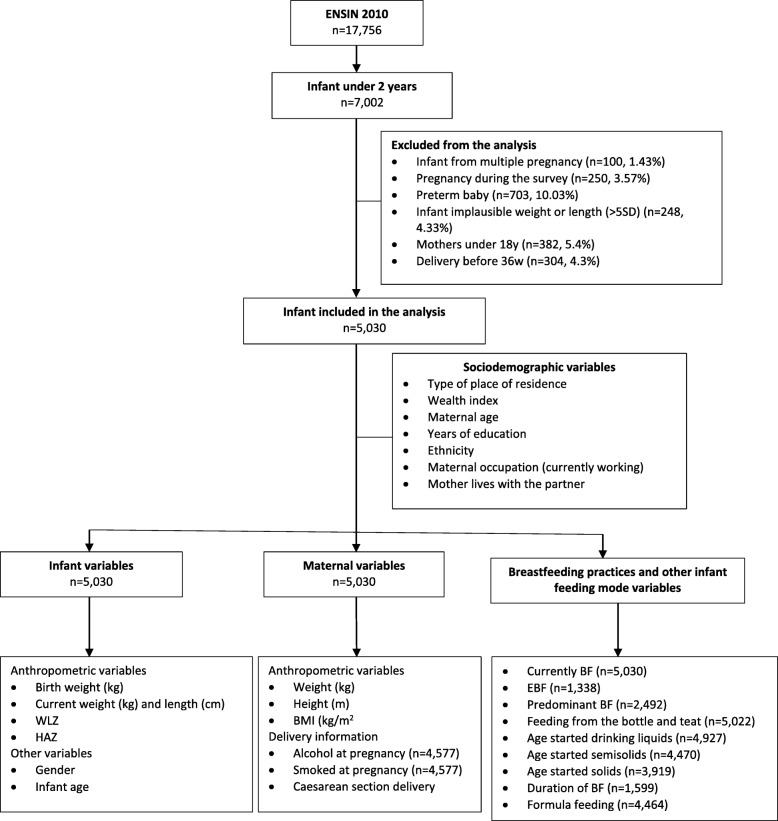
Sample analysed and study variables flow diagram. Secondary analysis, ENSIN 2010

An overall sample of *n* = 5030 mother-infant dyads with complete anthropometric measures was analysed. Information collected in the survey about EBF (only in infants under 6 months), current BF and predominant BF were available for *n* = 1338, n = 5030 and *n* = 2492 subjects respectively (Fig. 1). 63.4% of the study population lived in urban areas and 63% were in the lowest socioeconomic group. The mean maternal age was 26.6 years (±6.6) and the mean maternal years of education were 8.4 (±3.9). The majority of the sample were mestizo (72%), not working (59.3%) and living with the partner (75.9%). During pregnancy, 9% consumed alcohol and 2.1% smoked. Caesarean section deliveries accounted for around one-third of the sample (31.4%). The mean birth weight and infants ages were 3.3 kg (±0.5) and 12.1 months (±7.1), respectively. 51.8% of the infants were male (Table 1).

**Table 1:** Baseline characteristics compared between WLZ categories, ENSIN 2010

	Overall (*n* = 5030)	WLZ					
		Wasting <−2 SD (*n* = 74)	Normal ≥ − 2 SD, ≤ + 2 SD (*n* = 4663)	Overweight > + 2 SD (*n* = 293)	OR	95% CI	*p* value
					/Difference		
Sociodemographic characteristics (n, %)							
Type of place of residence							
Rural	1841 (36.6)	27 (36.5)	1706 (36.6)	108 (36.9)	0.98 ^b^	0.77, 1.26	0.92
Urban	3189 (63.4)	47 (63.5)	2957 (63.4)	185 (63.1)	0.99 ^a^	0.62, 1.60	0.98
Wealth index							
Poorer and poorest	3167 (63)	54 (73)	2937 (63)	176 (60.1)	1.35 ^a^	0.72, 2.53	0.35
					1.03 ^b^	0.75, 1.43	0.82
Middle	945 (18.8)	12 (16.2)	882 (18.9)	51 (17.4)	1		
Richer and richest	918 (18.3)	8 (10.8)	844 (18.1)	66 (22.5)	0.54 ^a^	0.26, 1.13	0.13
					1.35 ^b^	0.92, 1.97	0.12
Maternal characteristics							
Age (y) (mean, SD)	26.6, (±6.3)	28 (±6.3)	26.6 (±6.3)	26.3 (±6.1)	−1.4 ^a^	−3.2, 0.35	0.16
					0.3 ^b^	−0.61, 1.21	0.99
Maternal education (y) (mean, SD)	8.4, (±3.9)	6.9 (±4.3)	8.3 (±3.9)	9.1 (±3.6)	1.4 ^a^	0.31, 2.54	0.01*
					−0.8 ^b^	−1.4, − 0.25	0.002*
Ethnicity (n, %)							
Minorities	1406 (28)	22 (29.7)	1294 (27.8)	90 (30.7)	0.92 ^a^	0.55, 1.51	0.69
Mestizo	3624 (72)	52 (70.3)	3369 (72.2)	203 (69.3)	1.15 ^b^	0.89, 1.49	0.27
Currently working (n, %)							
Yes	2047 (40.7)	29 (39.2)	1888 (40.5)	130 (44.4)	0.94 ^a^	0.59, 1.50	0.81
No	2983 (59.3)	45 (60.8)	2775 (59.5)	163 (55.6)	1.17 ^b^	0.92, 1.48	
Mother lives with the partner (n, %)							
Yes	3816 (75.9)	56 (75.7)	3527 (75.6)	233 (79.5)	0.99 ^a^	0.58, 1.69	0.53
No	1214 (24.1)	18 (24.3)	1136 (24.4)	60 (20.5)	1.25 ^b^	0.93, 1.67	0.13
Pregnancy conditions							
Alcohol at pregnancy (*n* = 4577) (n, %)		n = 74	n = 4577	*n* = 291			
Yes	451 (9)	6 (8.1)	412 (9)	33 (11.3)	0.87 ^a^	0.38, 2.03	0.48
No	4491 (89.3)	68 (91.9)	4165 (91)	258 (88.7)	1.29 ^b^	0.88, 1.88	0.18
Smoked at pregnancy (n = 4577) (n, %)		n = 74	n = 4577	n = 291			
Yes	105 (2.1)	3 (4.1)	95 (2.1)	7 (2.4)	1.97 ^a^	0.61, 6.37	0.21
No	4837 (96.2)	71 (95.9)	4482 (97.9)	284 (97.6)	1.16 ^b^	0.53, 2.52	0.70
Caesarean section delivery (n, %)							
Yes	1579 (31.4)	21 (28.4)	1441 (30.9)	117 (39.9)	0.86 ^a^	0.52, 1.44	0.62
No	3451 (68.6)	53 (71.6)	3222 (69.1)	176 (60.1)	1.49 ^b^	1.17, 1.89	< 0.001**
Infant caractetistics							
Birth weight (kg)(*n* = 3877) (mean, SD)		*n* = 53	*n* = 3585	*n* = 239			
	3.3, (±0.5)	3.2 (±0.6)	3.3 (±0.5)	3.4 (±0.5)	0.08 ^a^	−0.08, 0.25	0.68
					−0.17 ^b^	−0.25, − 0.09	0.001**
Gender (n, %)							
Male	2605 (51.8)	40 (54.1)	2416 (51.8)	149 (50.9)	0.91 ^a^	0.57, 1.44	0.73
Female	2425 (48.2)	34 (45.9)	2247 (48.2)	144 (49.1)	0.96 ^b^	0.76, 1.21	0.75
Infant age (months) (mean, SD)	12.1, (±7.1)	8.9 (±6.9)	12.3 (±7.1)	11.1 (±6.9)	3.39 ^a^	1.41, 5.37	0.001**
					1.14 ^b^	0.12, 2.15	0.02*

Comparisons performed with χ^2^ test or Kruskall-Wallis test (all the continuous variables non-parametric distributed) as appropriate

^a^ Comparisons between normal and wasting

^b^ Comparisons between normal and overweight

* Significance at the level of *p* < 0.05

** Significance at the level of *p* < 0.001

### Infant anthropometric status, maternal BMI and breastfeeding practices

The prevalence of wasting, overweight and stunting was 1.5% (*n* = 74/5030), 5.8% (*n* = 293/5030) and 12.8% (*n* = 645/5030) respectively. Mean maternal weight, height and BMI was 59.8 ± 12 kg, 1.55 ± 0.06 m and 24.8 ± 4.6 kg/m^2^ respectively. EBF prevalence in infants under 6 months was 34.4%; other infant feeding mode variables analysed were current BF (68%), predominant BF (5.1%), duration of BF (mean = 7.9 ± 5.1 months), age of initiation of liquids, semisolids and solids (2.6 ± 2.5 months, 4.9 ± 2.1 months and 7.5 ± 2.5 months, respectively) and formula feeding (42%) (Table 2).

**Table 2:** Maternal anthropometry and breastfeeding practices compared between WLZ categories, ENSIN 2010

	Overall (*n* = 5030)	WLZ					
		Wasting <−2 SD (*n* = 74)	Normal ≥ − 2 SD, ≤ + 2 SD (*n* = 4663)	Overweight > + 2 SD (*n* = 293)	OR /Difference	95% CI	*p* value
Maternal anthropometry (mean, median, SD)							
Weight (kg)(*n* = 4750)		*n* = 165	*n* = 4496	*n* = 89			
	59.8, 57.6 (±12.0)	53.9, 52.5 (±10.9)	59.9, 57.7 (±11.9)	67.8, 65.8 (±12.7)	1.35 ^a^	−2.2, 4.9	0.99
					−4.1 ^b^	−5.8, −2.3	< 0.001**
Height (mt)(*n* = 4749)		n = 165	*n* = 4495	n = 89			
	1.55, 1.55 (±6.3)	1.51, 1.50 (±6.3)	1.55, 1.55 (±6.2)	1.57, 1.57 (±5.5)	0.72 ^a^	−2.58, 1.13	0.99
					−0.56 ^b^	−3.34, 0.76	0.39
BMI (kg/mt^2^)(*n* = 4747)		n = 165	*n* = 4493	n = 89			
	24.75, 24.1 (±4.6)	23.5, 22.8 (±4.3)	24.7, 24.1 (±4.6)	27.2, 26.6 (±4.8)	0.39 ^a^	−0.98, 1.75	0.99
					−1.47 ^b^	− 2.14, −0.79	< 0.001**
Breastfeeding practices and other infant feeding mode variables							
Currently BF (n, %)							
Yes	3419 (68)	60 (81.1)	3152 (67.6)	207 (70.6)	0.19 ^a^	0.14, 0.27	0.02*
No	1611 (32)	14 (18.9)	1511` (32.4)	86 (29.4)	1.15 ^b^	0.89, 1.49	0.27
EBF in 0 to 6 months (n, %)(n = 1338)		*n* = 34	*n* = 1211	*n* = 93			
Yes	460 (34.4)	15 (44.1)	410 (33.9)	35 (37.6)	1.54 ^a^	0.77, 3.1	0.21
No	878 (65.6)	19 (66.1)	801 (66.1)	58 (62.4)	1.18 ^b^	0.76, 1.82	0.45
Predominant BF (n, %)(n = 2492)		*n* = 36	*n* = 2313	*n* = 143			
Yes	127 (5.1)	2 (5.6)	118 (5.1)	7 (4.9)	1.09 ^a^	0.26, 3.62	0.71
No	2365 (94.9)	34 (94.4)	2195 (94.9)	136 (95.1)	1.04 ^b^	0.49, 2.18	0.55
Duration of BF (months) (mean, SD)(*n* = 1599)		n = 38	*n* = 1526	n = 35			
	7.9, (±5.1)	6.9, 7 (±4.5)	8, 7 (±5.1)	5.9, 6 (±4.4)	3.3 ^a^	−0.20, 6.81	0.07
					0.29 ^b^	−0.34, 0.38	0.99
Age of introduction of liquids including formula (months) (mean, SD)(*n* = 4927)		n = 176	*n* = 4659	*n* = 92			
	2.6, (±2.5)	2.5, 2 (±2.6)	2.6, 2 (±2.5)	2.4, 2.5 (±2.5)	0.75 ^a^	0.06, 1.45	0.03*
					0.02 ^b^	−0.34, 0.38	0.99
Age of introduction of semisolids (months) (mean, SD)(*n* = 4470)		*n* = 149	*n* = 4236	*n* = 85			
	4.9, (±2.1)	5.1, 5 (±2.6)	4.9, 5 (±2.1)	4.9, 5 (±1.7)	0.71 ^a^	0.05, 1.37	0.03*
					0.07 ^b^	−0.25, 0.39	0.99
Age of introduction of solids (months) (mean, SD)(*n* = 3919)		*n* = 127	*n* = 3713	*n* = 79			
	7.5, (±2.5)	7.7, 8 (±2.9)	7.5, 7 (±2.5)	7.5, 7 (±2.4)	0.53 ^a^	−0.35, 1.4	0.44
					0.11 ^b^	−0.3, 0.53	0.99
Formula feeding (n, %)(*n* = 4464)		*n* = 67	*n* = 4141	*n* = 256			
Yes	1877 (42)	34 (45.9)	1737 (41.9)	111 (43.4)	0.95 ^a^	0.58, 1.53	0.90
No	2587 (58)	40 (54.1)	2404 (58.1)	145 (56.6)	1.06 ^b^	0.82, 1.37	0.69

Comparisons performed with χ^2^ test or Kruskall-Wallis test or ANOVA (maternal height) as appropriate

^a^ Comparisons between normal and wasting

^b^ Comparisons between normal and overweight

* Significance at the level of p < 0.05

** Significance at the level of p < 0.001

### Predictors of wasting

The bivariate analysis showed that wasted infants were more likely not to be currently BF (OR = 0.19; 95% CI = 0.14, 0.27), to have an earlier age of introduction of liquids (Mean dif. = 0.75 months; 95% CI = 0.06, 1.45) and a later age of introduction of semisolids (Mean dif. = 0.71 months; 95% CI = 0.05, 1.37) when compared with infants with normal WLZ. Wasting was not predicted by maternal anthropometry, EBF or predominant BF variables; although there was a trend for infants under 6 months who were not EBF to have a higher prevalence of wasting (*n* = 19, 66.1% in non-EBF vs *n* = 15, 44.1% in EBF). The mean duration of BF was not significantly different between those who were wasted and those who were not (6.9 months v 8.7 months, mean dif. = 3.3 months; 95% CI = -0.2, 6.81). Regarding baseline characteristics, infants with wasting had mothers with fewer years of education (Mean dif = 1.4; 95% CI = 0.31, 2.54), and mothers with lower age (Mean dif = 3.39; 95% CI = 1.41, 5.37) (Table 2). Wealth index, a potential predictor of wasting, was not significantly associated; however, there was a trend for the poorer/poorest category to have a higher prevalence of wasting when compared with normal WLZ and overweight (*n* = 54, 73% for wasting; *n* = 2937, 63% for normal WLZ and *n* = 176, 60.1% for overweight) (Table 1). The unadjusted logistic regression model showed that current BF, age of introduction of liquids and semisolids were not significant predictors of wasting. In the final model, the only significant predictor of wasting was fewer years of maternal education (β = − 0.09; OR = 0.91; 95%CI = 0.86, 0.97); however, the model only predicted 0.4 to 1% of wasting in this population (Table 3).

**Table 3:** Overall logistic regression analysis for wasting, overweight and stunting, ENSIN 2010

	Unadjusted model					Adjusted model					Final model				
	β	OR	95% CI	*p* value	R^2^	β	OR	95% CI	*p* value	R^2^	β	OR	95% CI	*p* value	R^2^
Wasting ^a^															
Constant	−3.4	na	na	< 0.001**	0.003–0.006	−2.6	na	na	< 0.001**	0.009–0.021	−3.4	na	na	< 0.001**	0.004–0.01
Predictors															
Currently BF (1 = yes)	−0.56	0.57	0.31, 1.05	0.07		− 0.29	0.75	0.38, 1.47	0.40						
Age of introduction of liquids (months)	−0.05	0.95	0.83, 1.09	0.47		−0.05	0.95	0.82, 1.08	0.43						
Age of introduction of semisolids (months)	−0.13	0.88	0.75, 1.02	0.10		−0.08	0.92	0.78, 1.08	0.32						
Covariates															
Maternal education (y)						−0.09	0.91	0.85, 0.97	0.005*		−0.09	0.91	0.86, 0.97	0.003*	0.004–0.028
Infant age (months)						−0.03	0.97	0.92, 1.01	0.21						
Overweight ^a^															
Constant	−4.3	na	na	< 0.001**	0.005–0.012	−5.99	na	na	< 0.001**	0.02–0.04	−6.5	na	na	< 0.001**	0.014–0.031
Predictors															
Maternal BMI (kg/m^2^)	0.06	1.06	1.03, 1.08	< 0.001**		0.05	1.05	1.02, 1.08	0.001**		0.05	1.05	1.03, 1.08	0.001**	
Covariates															
Maternal education (y)						0.05	1.05	1.02, 1.09	0.005*		0.06	1.06	1.02, 1.10	0.001**	
Delivery by caesarean section (no)						−0.25	0.78	−0.59, 1.04	0.089						
Birth weight (kg)						0.001	1.001	1, 1.001	< 0.001**		0.001	1.001	1.00, 1.001	< 0.001**	
Infant age (months)						−0.01	0.98	0.96, 1.01	0.14						
Stunting ^b^															
Constant	−1.9	0.14	na	< 0.001**	0.01–0.02	1.42	4.14	na	0.23	0.05–0.11	1.96	7.1	na	< 0.001**	0.04–0.09
Predictors															
Maternal BMI (kg/m^2^)	− 0.03	0.97	0.94, 1.00	0.08		0.004	1.00	0.96, 1.05	0.84						
Duration of BF (months)	0.05	1.05	1.02, 1.08	0.003*		0.03	1.03	0.99, 1.08	0.17						
Age of introduction of liquids (months)	0.03	1.03	0.96, 1.11	0.39		−0.001	0.99	0.91, 1.10	0.99						
Age of introduction of semisolids (months)	0.05	1.05	0.95, 1.18	0.33		0.03	1.03	0.89, 1.20	0.69						
Age of introduction of solids (months)	−0.002	0.99	0.92, 1.08	0.96		0.03	0.96	0.87, 1.07	0.51						
Covariates															
Type of place of residence (urban/rural)						−0.32	0.72	0.41, 1.26	0.25						
Wealth index poorest						0.24	1.27	0.75, 2.16	0.37						
Maternal education (y)						−0.08	0.92	0.87, 0.98	0.01*		−0.09	0.92	0.89, 0.94	< 0.001**	
Etnicity (mestizo)						−0.38	0.68	0.42, 1.11	0.12						
Birth weight (kg)						−0.001	0.99	0.99, 1.00	< 0.001**		−0.001	0.99	0.99, 0.99	< 0.001**	
Gender (male)						−0.85	0.43	0.28, 0.66	< 0.001**		−0.47	0.62	0.49, 0.78	< 0.001**	
Infant age (months)						0.08	1.08	1.03, 1.14	0.001**		0.05	1.05	1.03, 1.07	< 0.001**	

^a^ Analysis performed with multinomial logistic regression, normal WLZ as reference catheogory

^b^ Analysis performed with binary logistic regression, normal LAZ as reference catheogory

* Significance at the level of *p* < 0.05

** Significance at the level of *p* < 0.001

### Predictors of overweight

Higher maternal BMI and higher maternal weight were significantly different (Mean dif = 1.5 kg/m^2^; 95% CI = 2.1, 0.8 and 4.1 kg; 95% CI = 5.8, 2.3, respectively) between infants with normal WLZ and those who were overweight, indicating a positive association between maternal BMI and weight, and infant overweight. Breastfeeding practices and other infant feeding mode variables were not significant predictors (Table 2). Overweight was also associated with more years of maternal education (Mean dif = 0.8 years; 95% CI = 1.4, 0.25), vaginal delivery (OR = 1.49; 95% CI = 1.2, 1.9), higher birth weight (Mean dif = 0.17 kg; 95% CI = 0.25, 0.09) and lower infant age (Mean dif = − 1.14 months; 95% CI = -0.12, − 2.1) (Table 1).

Higher maternal BMI was significantly related to infant overweight in the unadjusted (β = 0.06; OR = 1.06; 95% CI = 1.03, 1.08), adjusted (β = 0.05; OR = 1.05; 95% CI = 1.02, 1.08) and final model (β = 0.05; OR = 1.05; 95% CI = 1.03, 1.08). The adjusted model showed that more years of maternal education (β = 0.06; OR = 1.05; 95% CI = 1.02, 1.10) and higher birth weight (β = 0.001; OR = 1.001; 95% CI = 1.00, 1.001) were significant independent predictors of overweight, explaining 4% of this category (R^2^ = 0.04). However, delivery by caesarean section and infant age were no longer related to overweight (Table 3).

### Predictors of stunting

Compared to non-stunted infants, those with stunting had mothers with lower weight (Mean dif. = − 4.5 kg; 95% CI = -3. 5, − 5.5), shorter height (Mean dif. = − 0.04 m; 95% CI = -0.03, − 0.04) and lower BMI (Mean dif. = − 0.61 kg/m^2^; 95% CI = -0.22, − 0.99); as well as a shorter duration of BF (Mean dif. = − 1.33; 95% CI = -2.06, − 0.60) and earlier age of initiation of liquids (Mean dif. = − 0.61 months; 95% CI = -0.82, − 0.40), semisolids (Mean dif. = − 0.58 months; 95% CI = -0.77, − 0.40) and solids (Mean dif. = − 0.61 months; 95% CI = -0.84, − 0.38) (Table 4). Infants who lived in rural areas (OR = 1.74; 95%CI = 1.47, 2.05), with lower socioeconomic status (OR = 1.96; 95% CI = 1.53, 2.49 for poorer/poorest), with a mother with less years of education (Mean dif. = − 2.1 years; 95% CI = -1.78, − 2.43), belonging to the mestizo ethnic group (OR = 2.2; 95% CI = 1.85, 2.61), with lower birth weight (Mean dif. = − 0.23 kg; 95% CI = -0.17, − 0.28), male (OR = 1.27; 95% CI = 1.08, 1.51) and higher age (Mean dif. = 2.34 years; 95% CI = 0.29, 2.77) were more likely to be stunted (Table 5). Maternal height, analysed as a continuous variable, was associated with infant stunting after controlling for potential confounders including as type of place of residence, wealth index, ethnicity and infant gender and age (Mean dif = 0.09 m; 95% CI = 0.8, 0.11; data not shown).

**Table 4:** Breastfeeding practices compared between LAZ categories, ENSIN 2010

	Overall (*n* = 5030)	LAZ				
		Stunting <− 2 SD (*n* = 645)	Normal ≥ − 2 SD (*n* = 4385)	OR /Difference	95% CI	*p* value
Maternal anthropometry (mean, SD)						
Weight (kg)(n = 4750)		*n* = 619	*n* = 4131			
	59.8, (±12.0)	55.8, 54.5 (±11.36)	60.30, 58.10 (±11.9)	4.5	3.5, 5.5	< 0.001**
Height (mt)(*n* = 5384)		*n* = 619	*n* = 4130			
	1.55, (±0.06)	1.51, 1.51 (±0.06)	1.55, 1.55 (±0.06)	0.04	0.03, 0.04	< 0.001**
BMI (kg/mt^2^) (*n* = 5384)		n = 619	*n* = 4128			
	24.8, (±4.6)	24.23, 23.63 (±4.5)	24.8, 24.1 (±4.6)	0.61	0.22, 0.99	0.003*
Breastfeeding practices and other infant feeding modes						
Currently BF (n, %)						
Yes	3419 (68)	434 (67.2)	2985 (68.1)	1.04	0.87, 1.23	0.64
No	1611 (32)	211 (32.7)	1400 (31.9)			
EBF (n, %)(n = 1338)		*n* = 110	*n* = 1228			
Yes	460 (34.4)	46 (41.8)	414 (33.7)	0.71	0.48, 1.05	0.09
No	878 (65.6)	64 (58.2)	814 (66.3)			
Predominant BF (n, %)(n = 2492)		*n* = 296	*n* = 2196			
Yes	127 (5.1)	20 (6.8)	107 (4.9)	0.74	0.48, 1.12	1.16
No	2365 (94.9)	276 (93.2)	2089 (95.1)			
Duration of BF (months) (mean, SD)(n = 1599)		*n* = 211	*n* = 1388			
	7.9, 7 (±5.1)	9.1, 9 (±5.2)	7.7, 7 (±5)	−1.33	−2.06, −0.60	< 0.001**
Age of introduction of liquids including formula (months) (mean, SD)(n = 4927)		*n* = 624	*n* = 4303			
	2.6, (±2.5)	3.1, 3 (±2.9)	2.5, 2 (±2.4)	−0.61	− 0.82, − 0.40	< 0.001**
Age of introduction of semisolids (months) (mean, SD)(n = 4470)		*n* = 574	*n* = 3896			
	4.9, (±2.1)	5.5, 6 (2.4)	4.9, 5 (±2)	−0.58	−0.77, − 0.40	< 0.001**
Age of introduction of solids (months) (mean, SD)(n = 3919)		*n* = 523	*n* = 3396			
	7.5, (±2.5)	8, 8 (±2.66)	7.4, 7 (±2.5)	−0.61	− 0.84, − 0.38	< 0.001**
Formula feeding (n, %)(n = 4464)		*n* = 550	*n* = 3914			
Yes	1887 (42)	216 (39.3)	1661 (42.4)	0.87	0.73, 1.05	0.16
No	2587 (58)	334 (60.7)	2253 (57.6)			

Comparisons were performed with χ^2^ test, t test (maternal height) or U-Mann Whitney test as appropriate

* Significance at the level of *p* < 0.05

** Significance at the level of < 0.001

**Table 5:** Baseline characteristics compared between LAZ categories, ENSIN 2010

	Overall (*n* = 5030)	LAZ				
		Stunting <−2 SD (*n* = 645)	Normal ≥ − 2 SD (*n* = 4385)	OR /Difference	95% CI	*p* value
Sociodemographic characteristics (n, %)						
Type of place of residence						
Rural	1841 (36.6)	311 (48.2)	1530 (34.9)	1.74	1.47, 2.05	< 0.001**
Urban	3189 (63.4)	334 (51.8)	2855 (65.1)			
Wealth index						
Poorer and poorest	3167 (63)	502 (77.8)	2665 (60.8)	1.96	1.53, 2.49	< 0.001**
Middle	945 (18.8)	83 (12.9)	862 (19.7)	1		
Richer and richest	918 (18.3)	60 (9.3)	858 (19.6)	0.73	0.51, 1.02	0.07
Maternal characteristics						
Age (y) (mean, SD)	26.6, ±6.3	26.9 (±6.6)	26.5 (±6.3)	−0.39	−0.92, 0.13	0.25
Maternal education (y) (mean, SD)	8.4, ±3.9	6.5 (±3.9)	8.6 (±3.9)	2.1	1.78, 2.43	< 0.001**
Ethnicity (n, %)						
Mestizo	3624 (72)	366 (56.7)	3258 (74.3)	2.2	1.85, 2.61	< 0.001**
Minorities	1406 (28)	279 (43.3)	1127 (25.7)			
Currently working (n, %)						
Yes	2047 (40.7)	293 (45.4)	1754 (40)	1.24	1.06, 1.47	0.009*
No	2983 (59.3)	352 (54.6)	2631 (60)			
Mother lives with the partner (n, %)						
Yes	3816 (75.9)	499 (77.4)	3317 (75.6)	1.1	0.90, 1.34	0.34
No	1214 (24.1)	146 (22.6)	1068 (24.4)			
Pregnancy conditions						
Alcohol at pregnancy (n, %)(*n* = 4942)		*n* = 625	*n* = 4317			
Yes	451 (9)	46 (7.4)	405 (9.4)	0.76	0.56, 1.05	0.1
No	4491 (89.3)	579 (92.6)	3912 (90.6)			
Smoked at pregnancy (n, %)(n = 4942)		*n* = 625	n = 4317			
Yes	105 (2.1)	13 (2.1)	92 (2.1)	0.97	0.54, 1.75	0.93
No	4837 (96.2)	612 (97.9)	4225 (97.9)			
Caesarean section delivery (n, %)						
Yes	1579 (31.4)	169 (26.2)	1410 (32.2)	0.75	0.62, 0.90	0.002*
No	3451 (68.6)	476 (73.8)	2975 (67.8)			
Infant charactetistics						
Birth weight (kg) (mean, SD)(n = 3877)	3.3, ±0.5	3.1 (±0.6)	3.3 (±0.5)	0.23	0.17, 0.28	< 0.001**
Gender (n, %)						
Male	2605 (51.8)	368 (57.1)	2237 (51)	1.27	1.08, 1.51	0.004**
Female	2425 (48.2)	277 (42.9)	2148 (49)			
Infant age (months) (mean, SD)	12.1, ±7.1	14.2 (±6.9)	11.9 (±7.0)	−2.34	−0.29, −2.77	< 0.001**

Comparisons were performed with χ^2^ test or U-Mann Whitney test (all the continuous variables non-parametric distributed) as appropriate

Reference for all comparisons was normal LAZ

* Significance at the level of *p* < 0.05.

** Significance at the level of *p* < 0.001.

The unadjusted binary logistic model showed that none of the anthropometric and other infant feeding mode variables were related to stunting, apart from a longer duration of BF (β = 0.05; OR = 1.05; 95% CI = 1.02, 1.08). When adjusted for covariates, duration of BF was no longer significant. In the final model, stunted infants had mothers with fewer years of education (β = − 0.09; OR = 0.92; 95% CI = 0.89, 0.94), lower birth weight (β = − 0.001; OR = 0.99; 95% CI = 0.99, 0.99), were female (β = − 0.47; OR = 0.43; 95% CI = 0.49, 0.78) and had higher infant age (β = 0.05; OR = 1.08; 95% CI = 1.03, 1.07) (Table 3). EBF in infants up to 6 months did not predict stunting (OR = 0.75, 95% CI = 0.14, 1.63; data not shown). The association between infant age and wasting, overweight and stunting was significant in the binary analyses, but only remained significant in the final model for stunting.

## Discussion

To our knowledge, this is the first study in Colombia that describes the associations between maternal BMI or infant feeding mode and the prevalence of infant wasting, overweight and stunting. The main finding was a positive association between maternal BMI and infant overweight after controlling for potential confounders. This finding is in accordance with studies carried out in Colombia in children aged 5 to 12 years that found a relationship between childhood obesity and maternal overweight (aOR = 2.22; 95% CI = 1.73, 2.85) and obesity (aOR = 3.6; 95% CI = 2.64, 4.93) [33], and with other Latinamerican studies that show a higher risk of child overweight (>85th percentile) or obesity (>95th percentile) in those with obese mothers (OR = 2; 95% CI = 1.25, 3.47 and OR = 2.14; 95% CI = 1.12, 4.08) [34]. Although it was not possible to evaluate the relationship between maternal weight during pregnancy and gestational weight gain or infant weight change with infant overweight, our results could be interpreted as suggesting an association between maternal and infant weight which could be initiated during pregnancy or even before conception [35, 36]. This may reflect a combination of environmental factors and shared genetic factors between mother and infant. In Hispanic mothers, a high maternal genetic obesity risk score is associated with increased fetal weight during the first trimester of pregnancy (β = 3.1 g, 95% CI = 1.1–5.1, *P* = 0.003 for interaction) [37] and the ancestral genetic background is associated with excess weight in infant under 12 mo (OR = 3.85; 95%CI = 1.92–7.70) [38].

The relationship between maternal anthropometry or infant feeding mode variables with stunting did not persist in multivariable analyses. However, the association of maternal height with infant stunting suggests that genetic factors may contribute, as reported in a study in Mexico where short maternal stature (< 1,45 m) predicted infant stunting (OR = 3.9; 95% CI = 3.2, 4.8). This association persisted only in some areas of Mexico after adjusting by area of residence, suggesting that a shared adverse environment could also be relevant [39]. Stunted infants were older, most likely due to the cumulative impact of adverse health and nutritional exposures in utero and during the first months of life that prevent the infant from attaining its linear growth potential [40].

Unexpectedly, EBF, BF duration, age of initiation of liquids, semisolids and solids and formula feeding were not associated with infant wasting. Cross-sectional studies have shown an inverse association between breastfeeding and the rate of wasting in low-income countries [41]; in our study we found the same trend between a higher prevalence of BF and lower prevalence of wasting which could be clinically important although not significant. Maternal recall bias, difficulties in evaluating and defining complex variables such as EBF and duration of BF could be reasons for the lack of associations with infant anthropometry. Also, the ENSIN survey only recorded information about EBF in infants up to 6 months, so there was a smaller sample for evaluation of the associations EBF and anthropometry. Although the data recorded in ENSIN is evaluated as high quality by DHS based on missing values and inconsistencies; this does not necessarily exclude inaccuracies in the data collected [42], mainly due to the measurement in the home which does not guarantee a flat surface, and the estimation of infant weight (measured with the mother).

A growing body of evidence shows the importance of maternal nutritional status, especially during the perinatal period, on infant nutritional status (The first 1000 days of life) [21, 35, 43]. This association has been reported in studies where prepregnancy BMI had a positive correlation with birth weight, length and head circumference (β = 0.274, 0.094 and 0.101, respectively; *p* < 0.05 in all cases) [44]. Also, poor maternal nutritional status predicts undernutrition in infants at 6 and 12 mo [45]. Our study suggests that the association between maternal BMI and infant nutritional status can persist up to 24 mo, and maternal BMI is an apparent influential modifiable factor that requires adequate pre and postnatal advice for women.

Maternal education was associated with infant wasting, overweight and stunting, as reported in some other studies where each additional level of education was associated with a 44% reduction in stunting [46]. In a pooled analysis of five DHS surveys carried out in Bangladesh using data from 1999 to 2011, a lower maternal education level was consistently associated with infant stunting, wasting and underweight across the time-period of study [47]. In low socioeconomic populations, maternal education was inversely associated with child overweight, after controlling for gender [48]. However, we found an increased risk of infant overweight in more highly educated mothers, defined in this cohort as completing elementary and secondary studies but not college or further education. As maternal education could be a proxy for socioeconomic status, this association merits further analysis, including environmental conditions and maternal health-seeking behaviour [49]. Maternal education could protect against infant wasting and stunting if public policy focuses on the empowerment of women and their role in infant development.

The birth weight reflects fetal development and was found to be a significant predictor of infant anthropometry in this secondary analysis. Although one limitation was the fact that birth weight was self-reported by the mother, there was a consistent, positive and strong association between birth weight and overweight and an inverse association with stunting. This finding is in agreement with other studies in which birth weight is associated not only with anthropometry later in life but also with the risk of metabolic syndrome during adolescence (OR = 1.4; 95%CI = 1.2–1.6) [50] and adulthood, regardless of the presence or absence of gestational diabetes [51].

This study had some limitations, such as the oversampling of mestizo subjects and those with lower and lowest socioeconomic status, which may limit the generalizability of the findings to other Colombian ethnic and socioeconomic groups. Also, the ENSIN 2010 survey did not collect information about important aspects that could influence infant nutritional status such as paternal anthropometric status, pre-conceptional maternal weight and maternal weight gain during pregnancy. Unfortunately, the EBF variable was constructed using the information on both infant food consumption from the previous 24 h and data on the age of initiation of liquids other than breast milk, semisolid and solid foods. Inconsistencies in the prevalence of EBF using both methods suggest that mothers may not have understood the concept of EBF or the questions were inadequate to explore this. BF support at health institutions in Colombia is not well described in the survey, and that lack of information did not allow us to explore associations between BF counselling and duration of BF and EBF. Another limitation was that ENSIN 2010, which provided data for this secondary analysis, is a cross-sectional survey which does not permit prospective analysis of the effect of the factors associated with infant anthropometry.

## Conclusion

Greater understanding of factors associated with infant anthropometry during the first 2 years of life is vital in order to influence the risk of both wasting and overweight. In the present secondary analysis, we found a strong association between higher maternal BMI and infant overweight, but no association with EBF, BF and age of initiation of liquids, semisolids and solids. Thus, modifiable factors related to maternal nutritional status such as maternal BMI and maternal weight, are important and plausible targets to develop public policy at the population level that promotes healthy growth in the infant population. Efforts in primary care health to promote healthy maternal weight, starting pre-conception, but continuing during pregnancy, may reduce the risk of overweight and obesity in the offspring and have long-term health benefits.

## Data Availability

The database is available at the Demographic and Health Surveys Program under permission of DHS.

## Abbreviations

BF: Breastfeeding
BMI: Body Mass Index
EBF: Exclusive Breastfeeding
ENSIN: Encuesta Nacional de la Situación Nutricional
g: Gram
kg: Kilogram
LAZ: Length for Age Z score
mo: Months
w: Week
WAZ: Weight for Age Z score
WHO: World Health Organization
WLZ: Weight for Length Z score
y: Years
